# Idiopathic granulomatous mastitis: imaging update and review

**DOI:** 10.1007/s13244-016-0499-0

**Published:** 2016-05-24

**Authors:** Robert T. Fazzio, Sejal S. Shah, Nicole P. Sandhu, Katrina N. Glazebrook

**Affiliations:** Department of Radiology, Mayo Clinic, 200 First St. SW, Rochester, MN 55905 USA; Department of Laboratory Medicine and Pathology, Mayo Clinic, 200 First St. SW, Rochester, MN 55905 USA; Department of Medicine, Mayo Clinic, 200 First St. SW, Rochester, MN 55905 USA

**Keywords:** Breast, Granulomatous mastitis, MRI, Breast ultrasound, Mammography

## Abstract

**Objectives:**

The purpose of this study was to review the imaging features of idiopathic granulomatous mastitis (IGM) with clinical and pathology correlation.

**Methods:**

With institutional review board (IRB) approval, a retrospective search of the surgical pathology database from January 2000 to July 2015 was performed. Clinical, imaging and histology findings were reviewed. Cases of granulomatous mastitis without a known source, diagnosed with percutaneous or surgical biopsy, were included in our analysis.

**Results:**

Seventeen cases of IGM were identified with imaging available for review. The majority of patients presented with a palpable abnormality, whereas a minority were asymptomatic with an abnormal screening mammogram. At imaging, IGM most often demonstrated a focal asymmetry at mammography, a hypoechoic mass with irregular or angular margins at ultrasound, and robust enhancement with mixed progressive and plateau kinetics at magnetic resonance imaging (MRI). Axillary lymph nodes were reactive in appearance at ultrasound. Molecular breast imaging performed in one case showed mild focal asymmetric radiotracer uptake.

**Conclusion:**

IGM is a rapidly progressive rare inflammatory condition of the breast resulting in non-necrotizing granuloma formation. Imaging features mimic breast carcinoma and diagnosis can be difficult. Radiologists’ awareness of this condition is essential to prevent delayed or unnecessary treatment.

**Teaching points:**

• *Idiopathic granulomatous mastitis is rapidly progressive inflammatory condition.*

• *Imaging features may mimic breast carcinoma or infection.*

• *Ultrasound shows irregular hypoechoic masses with increased vascularity and sinus tracts.*

• *MRI shows irregular, enhancing masses or non-mass enhancement with microabscesses.*

• *MRI is useful for assessment of breast involvement and response to treatment.*

## Introduction

**Fig. 1:**
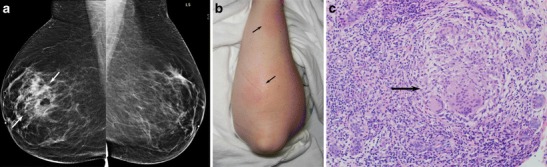
A 27-year-old woman with an enlarging palpable right breast mass admitted to the hospital for intractable joint pain. An erythematous rash was noted on her right breast and extremities. She was 8 months post-partum, breastfeeding with difficulty on the right. Bilateral mediolateral oblique mammogram (**a**) demonstrates regional asymmetry in the middle depth upper breast (*arrows*). **b** Clinical photograph of the patient’s forearm shows erythematous areas (*arrows*), representing erythema nodosum. **c** Photomicrograph (original magnification ×200; haematoxylin-eosin [H-E] stain) showing non-necrotizing granulomatous inflammation (*arrow*) composed of histiocytes and giant cells

Idiopathic granulomatous mastitis (IGM) is a rare, benign inflammatory condition of the breast first reported by Kessler and Wolloch in 1972 [1]. It occurs primarily in women of childbearing age, most often in postpartum or breastfeeding mothers. The etiology of IGM remains elusive. Reports have suggested an association with autoimmune disease or the result of a directed response to trauma, metabolic or hormonal processes [2–5]. An association with *Corynebacterium* species has also been proposed [6, 7], though by definition, diagnosis necessitates exclusion of bacteria from microbiology and histology specimens. Histologically, IGM is characterized by non-necrotizing granuloma formation with a localized infiltrate of multi-nucleated giant cells, plasma cells, epithelioid histiocytes and lymphocytes [1, 2, 5, 8–10] (Fig. 1). A neutrophilic infiltrate may also occur with formation of organized microabscesses [2, 5].

Imaging demonstrates a varied appearance based on the timing of radiographic evaluation, extent of inflammation and possibility of prior intervention [4, 8, 11–15]. IGM is frequently aggressive and typically demonstrates features of infectious mastitis or inflammatory breast carcinoma. Diagnosis of IGM can be difficult and is frequently delayed. Malignancy and all other possible causes of mastitis must be excluded before a diagnosis of IGM can be considered. Once diagnosed, treatment is often difficult and prolonged. Current treatment strategies favor conservative approaches with surgery reserved for refractory or aggressive cases [3, 16–22]. However, recurrence is unpredictable and may result regardless of treatment strategy. In this article, we discuss the histopathology, pathogenesis and clinical manifestations of IGM; provide an imaging update of this rare inflammatory condition through illustration of cases at mammography, ultrasound, magnetic resonance imaging (MRI) and molecular breast imaging (MBI); and discuss diagnosis, treatment approaches and outcomes.

## Materials and methods

With institutional review board (IRB) approval, a search of surgical pathology records of approximately 15,500 breast biopsies performed at our institution from January 2000 to July 2015 yielded 17 cases of IGM with imaging available for review. Clinical records, imaging and pathology specimens were reviewed by subspecialists in clinical breast health, breast radiology and breast pathology. Cases of IGM identified in the study time frame were diagnosed with image-guided core needle biopsy or surgical excisional biopsy. All cases of granulomatous mastitis without an identifiable etiology were included in this review; cases of mastitis attributed to autoimmunity, infection, trauma, metabolic, or hormonal processes were excluded. An additional case of granulomatous mastitis identified in a 34-year-old pregnant patient is included as a companion case, as one of multiple diagnostic/therapeutic breast aspirations grew Corynebacterium kroppenstedtii from the aspirated material.

## Results

**Fig. 2:**
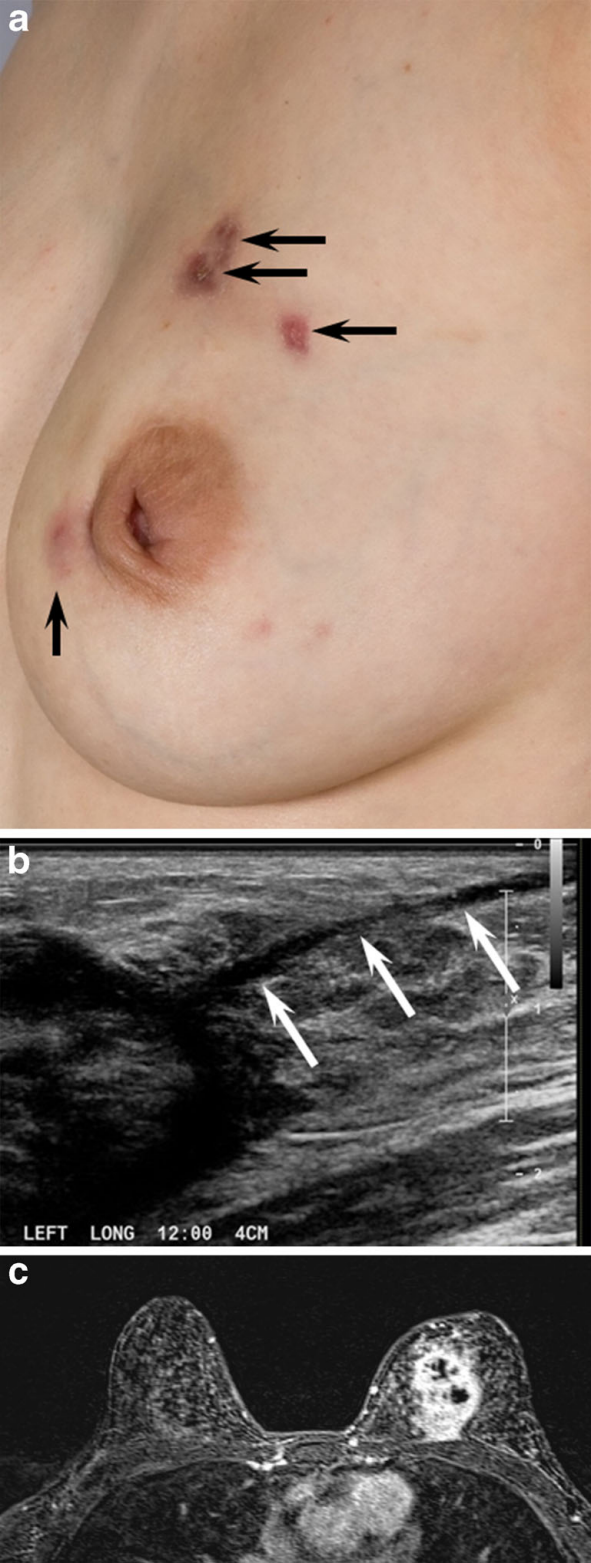
A 24-year-old Albanian mother of a 4-year-old (breastfed for 1 year) presented with a left breast lump, pain, erythema and drainage from multiple sites. Clinical photograph (**a**) of the left breast shows four eschars (*arrows*), two of which in the upper breast were draining cloudy fluid. Also shown is nipple inversion. **b** Ultrasound (US) image of the upper left breast shows a hypoechoic mass with indistinct margins with tract extending into adjacent tissue and to the skin surface (*arrows*) to the uppermost eschars noted the clinical photograph (**a**). **c** Axial T1-weighted post-contrast subtraction axial MRI image shows an irregular, intensely enhancing mass in the left breast extending to the chest wall with central areas of non-enhancement consistent with abscess formation. Ultrasound biopsy revealed IGM

**Fig. 3:**
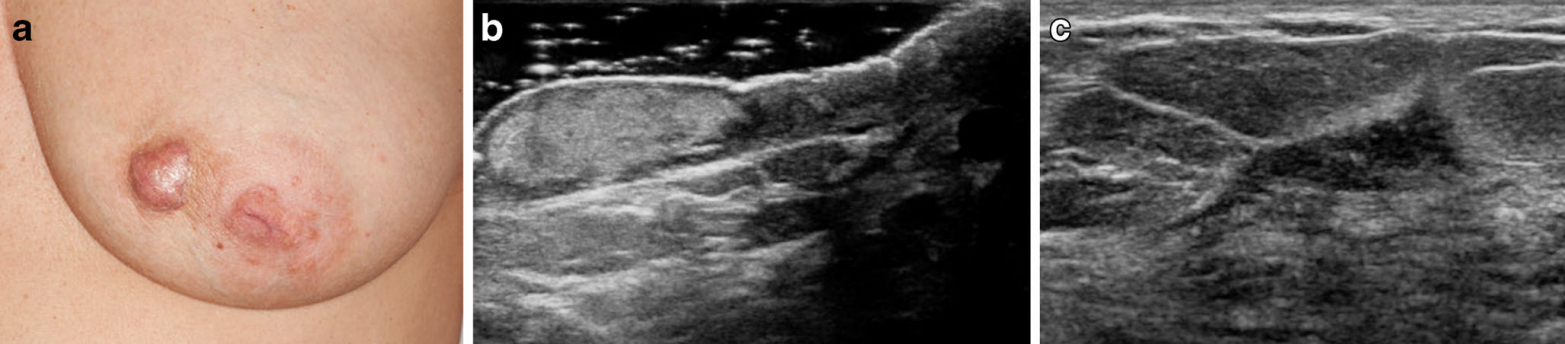
A 50-year-old woman presenting with painful, rapidly enlarging areolar skin lesion and breast mass. Clinical photograph (**a**) of the left breast shows a raised red skin lesion at the 9 o’clock position of the areola. Skin biopsy showed non-necrotizing granulomatous inflammation. **b** US image of the left areola and nipple show a hyperechoic area of skin thickening, which involves the dermis but does not connect to the underlying breast tissue. **c** US of the palpable breast mass demonstrates an irregular hypoechoic mass with angular margins and hyperechoic rim which did not connect to the areolar skin lesion. Percutaneous biopsy showed non-necrotizing granulomatous inflammation. Special stains for microorganisms were negative

**Fig. 4:**
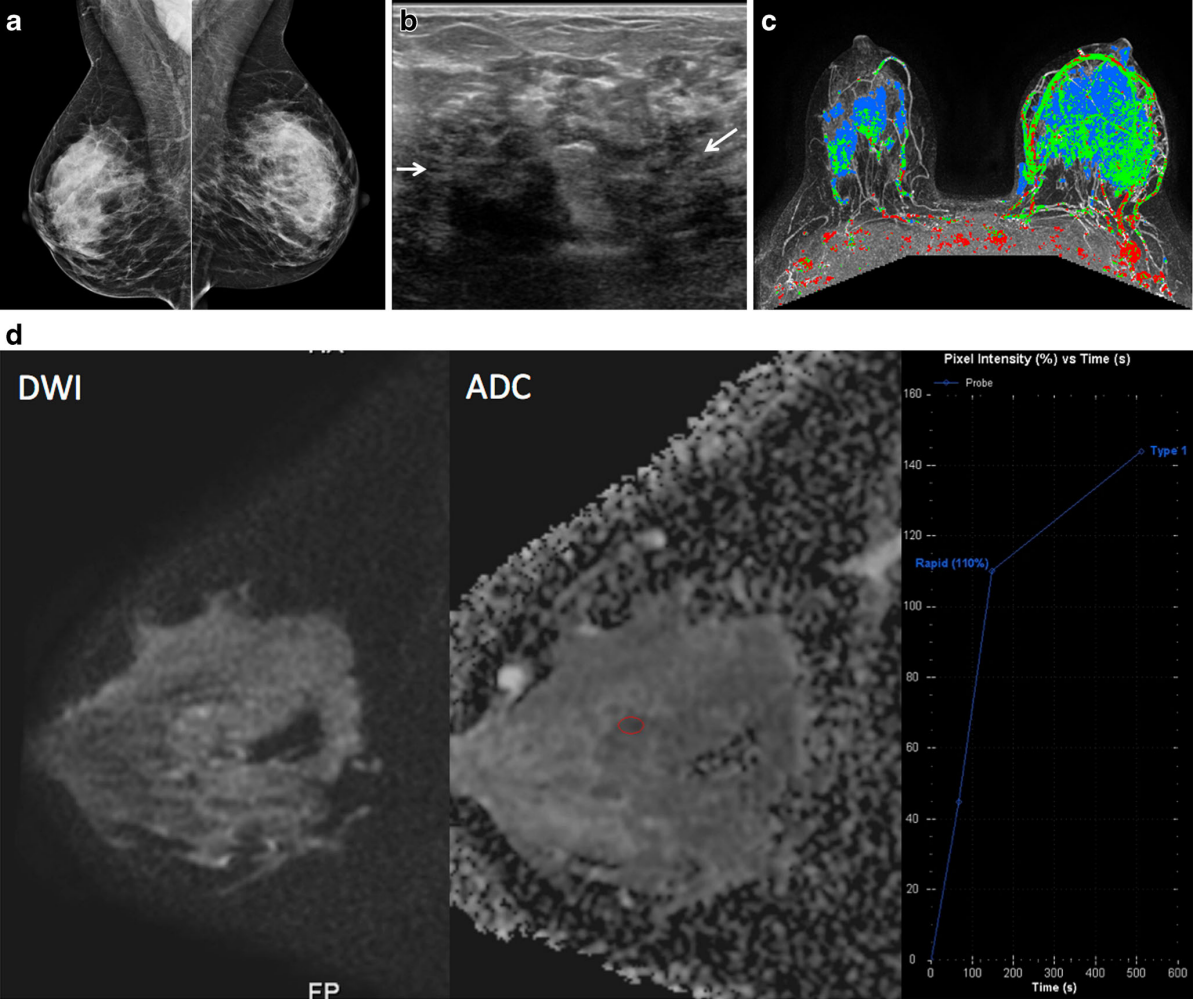
A 36-year-old female mother of two with a left breast lump and bilateral breast pain; IGM affecting both breasts. This patient breastfed both children; the last time was 2 years prior to presentation. **a** Bilateral mediolateral oblique mammograms show heterogeneously dense breast tissue but no abnormality in the left upper breast in the region of the palpable mass. **b** US image of the left upper outer breast shows a mixed echogenicity shadowing mass with indistinct margins (*arrows*), corresponding to a region of patient concern. **c** Axial T1 weighted post-contrast MIP with color kinetic analysis demonstrates bilateral heterogeneous enhancement, left greater than right, showing extent of involvement in both breasts (blue represents progressive enhancement and green plateau enhancement). **d** Sagittal diffusion-weighted (b = 800) image (*left*), corresponding ADC map (centre; 0.9 × 10^−3^ mm^2^/s), and time intensity enhancement curve (*right*) of the right breast demonstrates moderate restricted water diffusion with lower mean ADC values than what is observed in normal breast tissue

The mean age of IGM diagnosis was 44 years (range 25 to 72). Thirteen patients were premenopausal, all with prior full term pregnancies. Four patients were postmenopausal. The most common clinical presentation was a new enlarging unilateral breast mass (*n* = 14) (Fig. 1). Two patients had either skin ulceration with draining sinus tracts (Fig. 2) or a raised skin lesion with associated palpable masses (Fig. 3). Less common complaints included pain, erythema, nipple discharge, nipple retraction and axillary fullness. Two patients had skin lesions seen on the upper extremities consistent with erythema nodosum (Fig. 1). Four patients were asymptomatic with an abnormality detected on screening mammography. All cases but one were unilateral; bilaterality was unexpectedly identified at MRI with ill-defined masses demonstrated in both breasts of a 36-year-old premenopausal patient (Fig. 4).

**Fig. 5:**
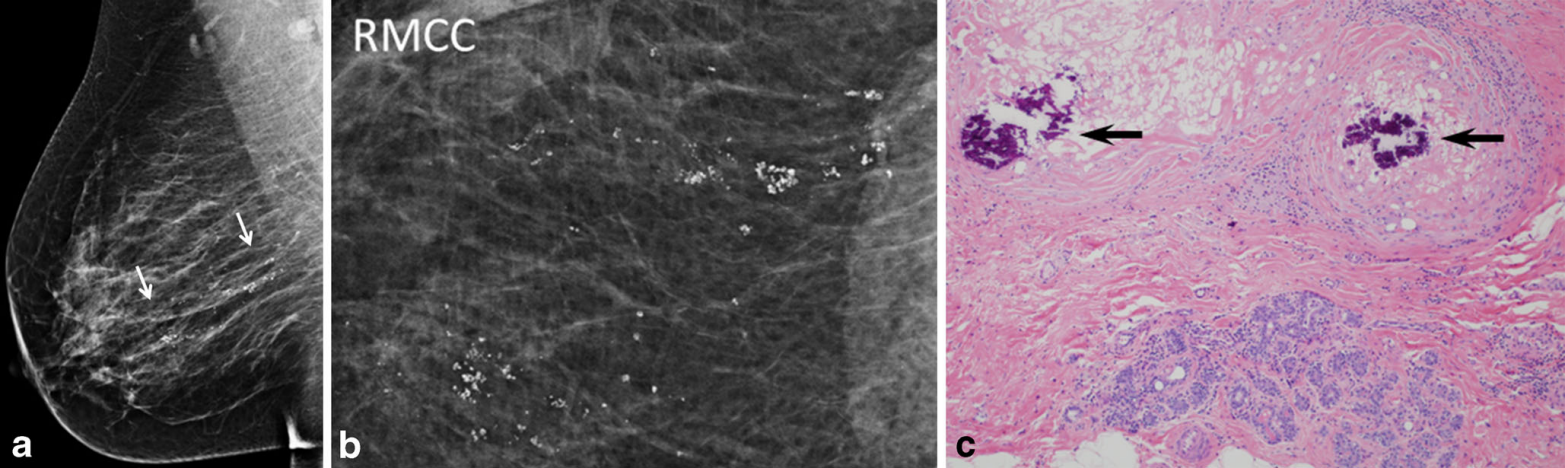
A 37-year-old asymptomatic mother of three who finished breastfeeding her third child 4 months prior to scheduled screening mammogram. **a** Unilateral right mediolateral oblique and **b** magnified craniocaudal mammograms demonstrate abundant segmental coarse heterogeneous calcifications not present on a prior comparison study performed 2 years earlier (not shown). **c** Photomicrograph (original magnification × 100; H-E stain) of stereotactic biopsy specimen demonstrates calcifications (*arrows*) in the background of histiocytic inflammation within a breast lobule

Mammography was available in 16 cases. IGM presented mammographically as unilateral focal or regional asymmetry in 12 patients (Fig. 1). There was associated architectural distortion and irregular mass in four cases. Interestingly, one case presented at screening mammography with segmental coarse heterogeneous calcifications (Fig. 5). Mammography failed to identify an abnormality in four patients (24 %), presumably due to overlapping dense breast parenchyma obscuring potential findings.

**Fig. 6:**
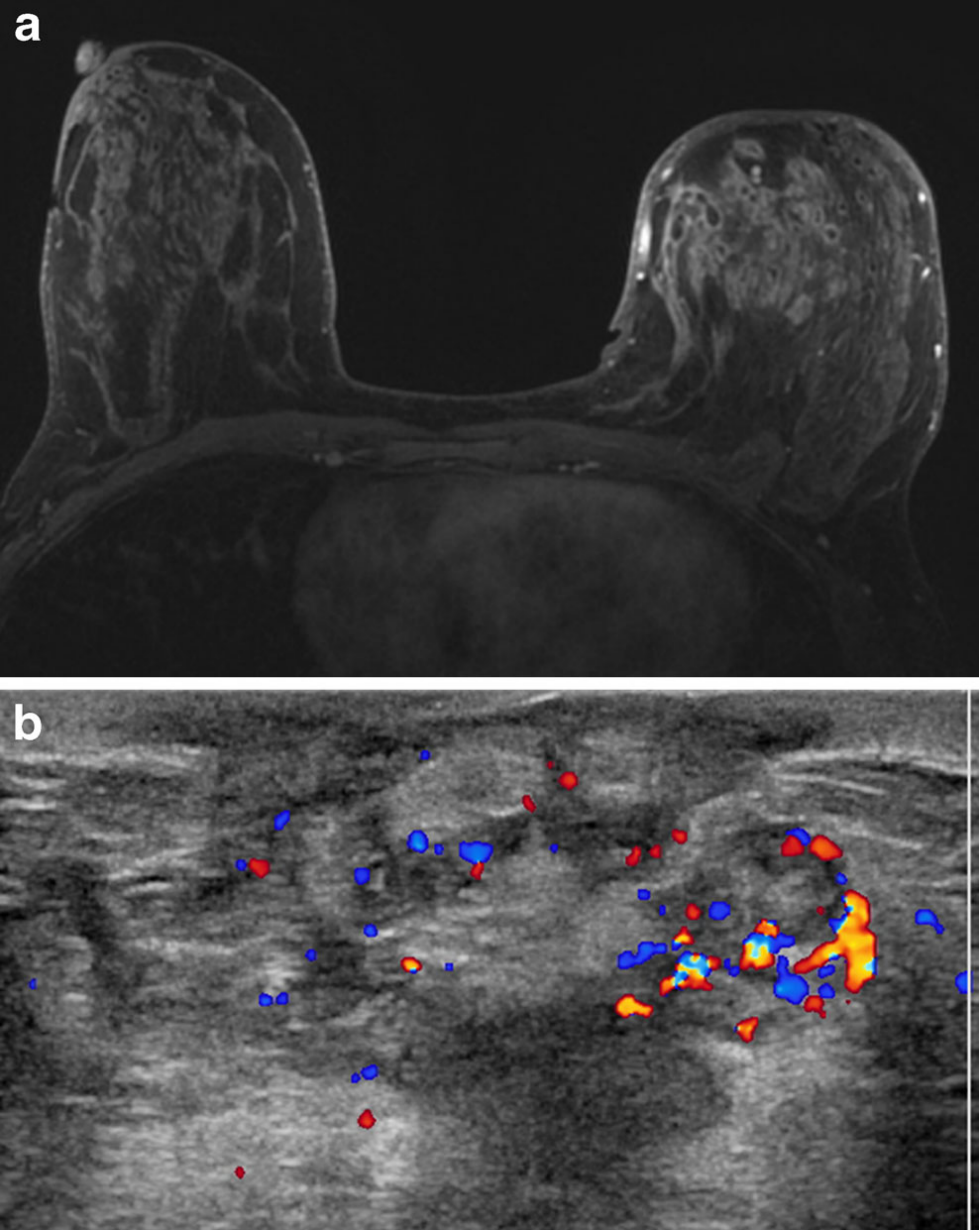
A 39-year-old woman treated with multiple prior incision and drainages of the lateral lower left breast, presenting with new pain and palpable thickening left inner upper breast. **a** Post-contrast, vibrant, T1-weighted, fat-saturated axial MRI image showing extensive rapid enhancement of the left inner breast with central areas of non-enhancement consistent with microabscess formation. **b** US of the same area shows multiple serpiginous hypoechoic areas with increased vascularity noted on color Doppler evaluation

Ultrasound (*n* = 15) demonstrated a hypoechoic mass(es) with indistinct, irregular or angular margins, hyperechoic rim and internal vascularity (Figs. 2, 3, 4 and 6). Posterior acoustic shadowing was noted in four cases (Fig. 4). Sonographically reactive appearing nodes were identified in the ipsilateral axilla of eight patients. Fine needle aspiration of axillary lymph nodes in three cases showed similar results: mixed lymphocytic population consistent with a reactive lymph node. Hypoechoic sinus tracts were noted to interleave between otherwise normal parenchyma, extending between masses or to the skin in seven cases (Fig. 2).

MRI (*n* = 5) demonstrated irregular enhancing masses, most with ill-defined margins and surrounding non-mass enhancement. Mixed progressive and plateau enhancement kinetics were observed in all cases (Figs. 2, 4 and 6), some interspersed with small regions of more rapid contrast enhancement and washout. Three advanced cases demonstrated T2 hyperintense, peripherally enhancing masses with central areas of non-enhancement representing abscess formation (Fig. 2). Enlargement of the affected breast with skin and nipple involvement was identified in one case (Fig. 4). Restricted diffusion (b = 0, b = 800) was also noted in regions of involvement (Fig. 4) with mean ADC values of 1.0 × 10^−3^mm^2^/s (adjacent normal parenchyma 2.3 × 10^−3^mm^2^/s). Axillary lymphadenopathy was not a prominent feature at MRI with reactive changes noted in the axillary nodes.

**Fig. 7:**
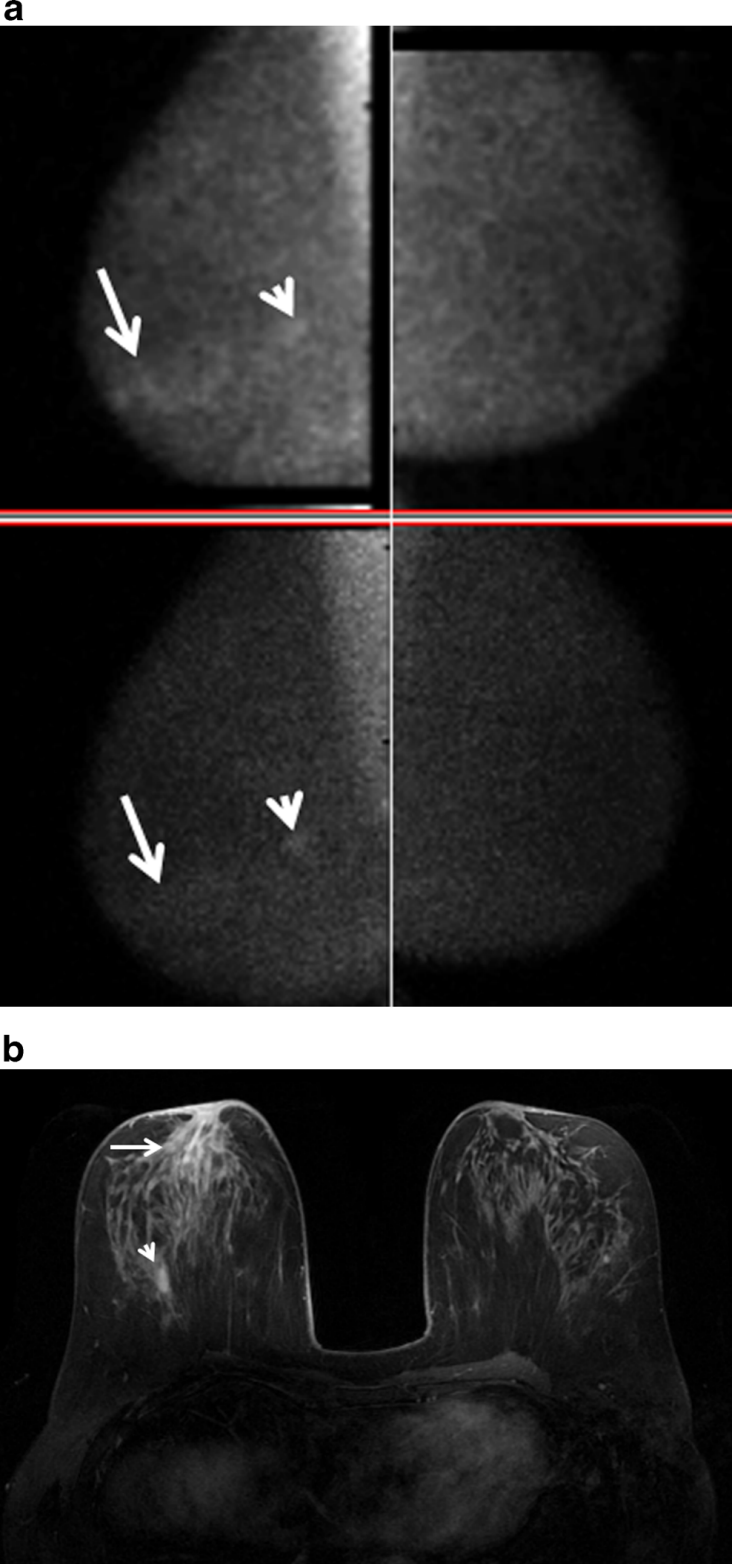
A 50-year-old woman with right breast pain and redness not resolving with antibiotics. Biopsy showed IGM. **a** Dual head prototype MLO bilateral molecular breast imaging (MBI) scanned prior to commercially available MBI demonstrates mild unilateral right non-mass subareolar uptake (*arrows*) best seen on the superior detector (*upper left image*). The posterior area of mass enhancement (*arrowhead*) was a biopsy proven fibroadenoma. Left breast is negative. **b** Axial T1 weighted post gadolinium fat saturated MRI image shows non-mass enhancement in the right subareolar region corresponding to the focal uptake on MBI (*arrows*); MR-guided biopsy revealed IGM. The enhancing fibroadenoma is noted posterior depth right breast (*arrowhead*)

MBI performed in one patient with unilateral breast pain demonstrated mild unilateral nonmass subareolar uptake corresponding to nonmass ductal enhancement noted on comparison MRI examination (Fig. 7).

Seven patients were treated with observation only, one had her oestrogen stopped, three were treated with prednisone and antibiotics, and one with repeated percutaneous drainage. Five patients proceeded to surgical excision following failure of conservative measures. Long-term follow-up was available in eight patients ranging from 1 to 12 years (mean 4 years). There were three patients with recurrences at 1, 5 and 7 years respectively. The first patient demonstrated clinical resolution initially but had recurrence of symptoms at 1 year, which resolved following surgical excision of the affected area. The second patient recurred 5 years following surgical excision. She was treated with observation alone, with resolution of symptoms within 6 months. The third patient was initially treated with surgical excision with a recurrence in the same breast 7 years later. She was subsequently treated with anti-inflammatories only and was then lost to follow-up.

## Discussion

IGM is a diagnosis of exclusion requiring careful histopathology review of biopsy specimens, as well as microbiological analysis. This rare inflammatory process is characterized by non-necrotizing granuloma formation in breast lobules (Fig. 1c). Necrosis is rare, a finding more typically seen with tuberculous mastitis [2, 10, 23, 24]. An associated inflammatory infiltrate composed of multi-nucleated giant cells, plasma cells, epithelioid histiocytes, and lymphocytes are typically isolated within affected lobules [4, 5, 10, 23] (Fig. 1c). Depending upon severity, this inflammatory response may extend into adjacent breast lobules. Involved parenchyma demonstrates loss of acinar structure and damaged ducts [2, 24]. A neutrophilic infiltrate and formation of sterile microabscesses may also be demonstrated [2, 19, 23].

The precise etiology of IGM is uncertain. Current theories of IGM etiology favor an inflammatory response within the connective tissue of breast stroma to glandular secretions leaked from damaged ductal epithelium [2]. Inflammation is localized within lobules and may result in a chemical mastitis. Potential precipitating factors include autoimmune disease, pregnancy and lactation, hyperprolactinemia, oral contraceptive use, trauma, and foreign body reaction, among others.

The autoimmune hypothesis is supported by the observation that some patients with IGM demonstrate extramammary manifestations of autoimmune disease such as inflammatory arthritis and erythema nodosum, exhibit a T-lymphocyte rich inflammatory infiltrate, and often respond favorably to treatment with corticosteroids [2, 8, 18, 25, 26] (Fig. 1). However, serological tests that are routinely positive in patients with autoimmune disease, such as rheumatoid factor (RF) and anti-nuclear antibody (ANA), demonstrate variable positivity in patients with IGM [8]. The association of pregnancy and lactation stems from the observation that the majority of women afflicted with IGM are premenopausal, as well as parous, and are either pregnant at the time of diagnosis or have given birth within 5 years of experiencing symptoms [23]. Nevertheless, postmenopausal patients have also been diagnosed with this condition, as seen in our series. Elevated prolactin levels (endogenous or exogenous source) as a potential trigger of IGM [5] is supported by the hypothesis that hyperprolactinemia may contribute to increased ductal secretions, leading to damaged ductal epithelium. An association with trauma has also been suggested [23], though difficult to substantiate. Interestingly, the majority of patients diagnosed with IGM are from developing countries. While this observation might reflect underdiagnosis of tuberculous mastitis [21], further investigation into the geographic disparity of this aggressive inflammatory disorder may be helpful towards elucidation of an etiology.

**Fig. 8:**
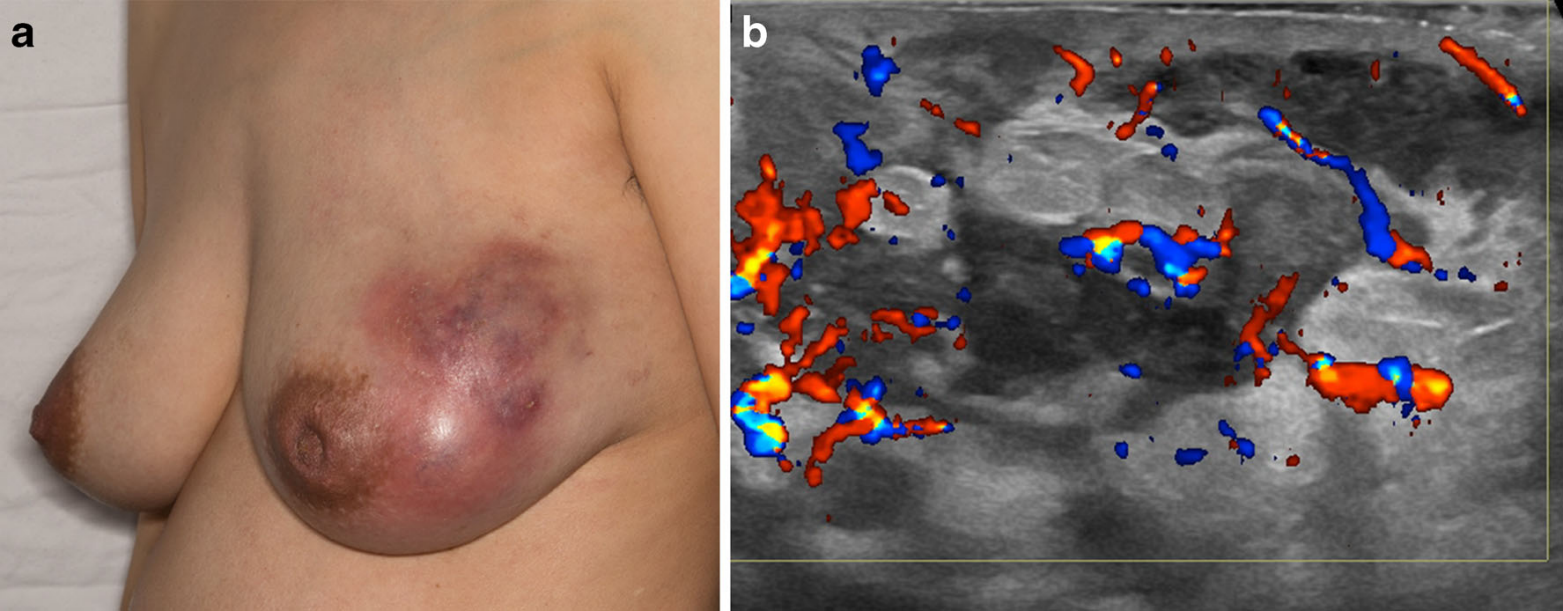
A 34-year-old pregnant patient with area of redness and pain left breast and associated nipple inversion. One of many aspirations grew Corynebacterium Kroppenstedtii. **a** Clinical photograph shows nipple retraction and a large area of inflammation involving the lateral left breast. **b** Ultrasound image of the left lateral breast demonstrates multiple hypoechoic masses with interconnecting tracts and marked parenchymal vascularity as seen on color Doppler evaluation, very similar to ultrasound findings in Fig. 6

Microorganisms must be absent from histopathology specimens and aspirates of affected tissue, including presumed abscess cavities. Gram stain, culture, and special stains including Zehl-Neelsen, Periodic acid-Schiff, GMS or other silver staining methods must be negative. Nevertheless, there have been a few reports of *Corynebacterium* species associated with tissue and fluid obtained from patients with presumed IGM [6, 7]. One pregnant patient from our series with clinical and imaging findings typical of granulomatous mastitis grew Corynebacterium kroppenstedtii from one of many otherwise sterile aspirations from her left breast. Importantly, her ultrasound findings were identical to cases of IGM from our study cohort, showing irregular hypoechoic masses with angular margins, communicating sinus tracts, and marked vascularity of the breast parenchyma on color Doppler evaluation (Fig. 8).

Symptoms in patients with IGM can include a palpable lump, localized or regional erythema, focal tenderness and peau d’orange [23, 27]. Nipple involvement is infrequent, but can include discharge [4, 12], scaling and retraction, with or without pain. Unilateral symptoms are most often reported; involvement of both breasts is less common [8]. The majority of patients are female, although cases have also been identified in males [10, 23, 28]. Most patients are premenopausal and report childbirth and breastfeeding within the previous 5 years; however, cases of IGM have been identified in patients as young as 11 and as old as 80 [27]. Occasionally, unilateral axillary lymphadenopathy is detectable on physical examination [22], a finding concerning for breast carcinoma with nodal metastatic disease. Eschars, ulceration and draining sinus tracts have been reported (Fig. 2). In some cases, sinus tracts may develop at the location of percutaneous core needle biopsy [8], presumably due to inadequate healing at the biopsy site. In advanced disease, the involved breast can be significantly enlarged and demonstrate skin retraction in more than one quadrant.

Mammographic findings in IGM include focal or regional asymmetry, a solitary mass or masses, skin thickening, skin and nipple retraction and axillary lymphadenopathy [4, 8, 11–15, 29]. In our series, the most common mammographic finding was a moderate-sized focal asymmetry corresponding to a region of palpable concern (Fig. 1). Regional asymmetry, trabecular thickening or multifocal involvement may also be noted in the affected breast. Skin thickening may occur, particularly when findings are noted in the anterior depth tissue. Nevertheless, mammography can also be normal, particularly in patients with dense breasts and/or a mild inflammatory response (Fig. 4). In many cases, the affected breast may appear larger than the contralateral breast, a finding most apparent on the full field mediolateral oblique view. Calcifications are exceedingly rare, but have been reported [4] in IGM (Fig. 5). Importantly, the mammographic appearance of IGM can be indistinguishable from invasive or inflammatory breast cancer prompting further imaging evaluation.

Ultrasound is helpful for evaluation of palpable abnormalities in patients with a normal mammogram (Fig. 4). Sonographic findings in IGM are variable and can include masses, architectural distortion, parenchymal edema, fluid collections, skin thickening and axillary lymphadenopathy [4, 8, 11–15, 29, 30]. The most common ultrasound findings are of a mixed echogenicity but predominantly hypoechoic mass with angular, irregular or indistinct margins, with or without sinus tracts insinuating into adjacent parenchyma (Figs. 2,4 and 6). There are variable degrees of posterior acoustic phenomena, including both enhancement and shadowing [8, 11, 31] (Fig. 4). Doppler imaging demonstrates increased internal blood flow within lesions and the surrounding breast parenchyma (Figs. 6 and 8) [4, 29]. In advanced cases, disease may present as fluid collections or abscess cavities, which can be aspirated for microbiological analysis [8]. Ultrasound is useful for documentation of sinus tracts extending to the skin surface, a finding that can be seen with delay in diagnosis or history of prior intervention (Fig. 2) [8, 12, 15]. Ultrasound is also helpful in evaluation of enlarged axillary lymph nodes, which most often demonstrate smooth reactive cortical thickening.

MR is a useful adjunct to mammography and ultrasound in evaluation of patients with IGM, indicated in patients with advanced, aggressive, or refractory disease. Importantly, MR provides the best estimate of disease extent and contralateral breast involvement. MRI findings in IGM are variable depending upon the severity of inflammation. Heterogeneous ill-defined masses and non-mass enhancement with mixed kinetics were identified in our series, as was noted in previously reported cases [8, 29–31] (Figs. 2 and 4). Often, progressive or plateau enhancement patterns predominate with interspersed areas of rapid enhancement and washout. Irrespective of kinetics, the affected parenchyma demonstrates intense enhancement compared to uninvolved tissue (Fig. 2). There may be associated peripherally enhancing masses with increased internal T2 signal, representing microabscesses [8]. In advanced disease, larger fluid collections can be seen interspersed within abnormal enhancement, with or without sinus tracts extending to the skin surface [8] (Figs. 2 and 6). Involved parenchyma displays restricted diffusion in the majority of cases (Fig. 4) with consistently lower mean ADC values (1.0 × 10^−3^mm^2^/s) than what is observed for normal breast parenchyma (2.3 × 10^−3^mm^2^/s). Our mean ADC values for IGM are close to those identified for suspicious breast lesions [32] and similar to what is reported for granulomatous inflammation of the prostate [33]. Presumably, the chronic inflammatory response in IGM results in reduced water diffusion capacity and decreased relative ADC values. Importantly, although ADC values in IGM are falsely positive for malignancy, time intensity curves are more benign, consistent with inflammation. Skin thickening is well demonstrated, as is rare involvement of the nipple and nipple areolar complex. Importantly, MR allows for assessment of disease progression or regression over time, potentially useful in difficult cases treated with conservative therapy or for documentation of recurrence.

Our solitary case with MBI demonstrated low-level nonmass uptake corresponding to mild nonmass enhancement identified on the comparison MRI (Fig. 7). Given the findings in our series, and additional evidence supporting the use of MBI to identify mammographically occult breast carcinoma, MBI would likely prove highly sensitive for detection of inflammatory uptake expected from IGM [34].

Because IGM mimics bacterial mastitis, commonly seen in young breastfeeding patients, early treatment with antibiotics is often employed, without success, leading to further workup. Surgical therapy, using wide local excision or mastectomy depending on extent of disease, was the treatment of choice in the 1970s and 1980s [21, 35, 36]. However, recurrence rates are high with surgery—approaching 25 %—particularly when excision of actively inflamed tissue is performed, often resulting in multiple re-operations to achieve cure [22, 37]. Recurrence may also be delayed, requiring additional treatment months after initial therapy. A hybrid approach has also been utilized with surgical excision performed after sufficient delay to allow for reduction in the degree of acute inflammation within the breast [12]. Conservative management has gained popularity in the last 20 years and is currently the preferred approach, with treatment options including close clinical surveillance, corticosteroid therapy, or treatment with direct immunomodulators such as methotrexate. Importantly, published reports using corticosteroids and immunomodulators for therapy include small sample sizes and there is currently no clinical consensus that these medications perform better than watchful waiting.

## Conclusion

IGM is a rare inflammatory condition of the breast that mimics infectious mastitis or inflammatory breast carcinoma. Clinical diagnosis is often one of exclusion, is delayed in the majority of cases, but should be facilitated by imaging. Contemporary breast imaging techniques are essential in documenting disease extent, providing guidance during percutaneous core needle biopsy and in helping to exclude malignancy. MR imaging can be useful in monitoring IGM behavior and clinical improvement, particularly in patients managed conservatively.

## References

[1] Kessler E, Wolloch Y (1972). Granulomatous mastitis: a lesion clinically simulating carcinoma. Am J Clin Pathol.

[2] Erhan Y, Veral A, Kara E (2000). A clinicopthologic study of a rare clinical entity mimicking breast carcinoma: idiopathic granulomatous mastitis. Breast.

[3] Akbulut S, Yilmaz D, Bakir S (2010). Methotrexate in the management of idiopathic granulomatous mastitis: review of 108 published cases and report of four cases. Breast J.

[4] Boufettal H, Essodegui F, Noun M (2012). Idiopathic granulomatous mastitis: a report of twenty cases. Diagn Interv Imaging.

[5] Lin C-H, Hsu C-W, Tsao T-Y, Chou J (2012). Idiopathic granulomatous mastitis associated with risperidone-induced hyperprolactinemia. Diagn Pathol.

[6] Taylor GB, Paviour SD, Musaad S (2003). A clinicopathological review of 34 cases of inflammatory breast disease showing an association between corynebacteria infection and granulomatous mastitis. Pathology.

[7] Mathelin C, Riegel P, Chenard M-P (2005). Granulomatous mastitis and corynebacteria: clinical and pathologic correlations. Breast J.

[8] Gautier N, Lalonde L, Tran-Thanh D (2013). Chronic granulomatous mastitis: imaging, pathology and management. Eur J Radiol.

[9] Lacambra M, Thai TA, Lam CCF (2011). Granulomatous mastitis: the histological differentials. J Clin Pathol.

[10] Tse GMK, Poon CSP, Ramachandram K (2004). Granulomatous mastitis: a clinicopathological review of 26 cases. Pathology.

[11] Han BK, Choe YH, Park JM (1999). Granulomatous mastitis: mammographic and sonographic appearances. AJR Am J Roentgenol.

[12] Hovanessian Larsen LJ, Peyvandi B, Klipfel N (2009). Granulomatous lobular mastitis: imaging, diagnosis, and treatment. AJR Am J Roentgenol.

[13] Memis A, Bilgen I, Ustun E (2002). Granulomatous mastitis: imaging findings with histopathologic correlation. Clin Radiol.

[14] Yilmaz E, Lebe B, Usal C, Balci P (2001). Mammographic and sonographic findings in the diagnosis of idiopathic granulomatous mastitis. Eur Radiol.

[15] Kocaoglu M, Somuncu I, Ors F (2004). Imaging findings in idiopathic granulomatous mastitis. J Comput Assist Tomogr.

[16] Erozgen F, Ersoy YE, Akaydin M (2010). Corticosteroid treatment and timing of surgery in idiopathic granulomatous mastitis confusing with breast carcinoma. Breast Cancer Res Treat.

[17] Konan A, Kalyoncu U, Dogan I (2012). Combined long-term steroid and immunosuppressive treatment regimen in granulomatous mastitis. Breast Care (Basel).

[18] Maffini F, Baldini F, Bassi F (2009). Systemic therapy as a first choice treatment for idiopathic granulomatous mastitis. J Cutan Pathol.

[19] Al-Khaffaf B, Knox F, Bundred NJ (2008). Idiopathic granulomatous mastitis: a 25-years experience. J Am Coll Surg.

[20] Kim J, Tymms KE, Buckingham JM (2003). Methotrexate in the management of granulomatous mastitis. ANZ J Surg.

[21] Bani-Hani KE, Yaghan RJ, Matalka II, Shatnawi NJ (2002). Idiopathic granulomatous mastitis: time to avoid unnecessary mastectomies. Breast J.

[22] Wilson JP, Massoll N, Copeland EM, Grobmyer SR (2007). Idiopathic granulomatous mastitis: in search of a therapeutic paradigm. Am Surg.

[23] Seo HRN, Na KY, Yim HE (2012). Differential diagnosis in idiopathic granulomatous mastitis and tuberculous mastitis. J Breast Cancer.

[24] Bakaris S, Yuksel M, Ciragil P (2006). Granulomatous mastitis including breast tuberculosis and idiopathic lobular granulomatous mastitis. Can J Surg.

[25] Lai ECH, Chan WC, Ma TKF (2005). The role of conservative treatment in idiopathic granulomatous mastitis. Breast J.

[26] Bes C, Soy M, Vardi S (2010). Erythema nodosum associated with granulomatous mastitis: report of two cases. Rheumatol Int.

[27] Patel RA, Strickland P, Sankara IR (2010). Idiopathic granulomatous mastitis: case reports and review of literature. J Gen Intern Med.

[28] Reddy KM, Meyer CER, Nakdjevani A, Shrotria S (2005). Idiopathic granulomatous mastitis in the male breast. Breast J.

[29] Al-Khawari HAT, Al-Manfouhi HA, Madda JP (2011). Radiologic features of granulomatous mastitis. Breast J.

[30] Dursun M, Yilmaz S, Yahyayev A (2012). Multimodality imaging features of idiopathic granulomatous mastitis: outcome of 12 years of experience. Radiol Med.

[31] Ozturk M, Mavili E, Kahriman G (2007). Granulomatous mastitis: radiological findings. Acta Radiol.

[32] Abowarda MH, Hasan DI, Elteeh OA (2015). Prepredictive value of ADC mapping in discriminating probably benign and suspicious breast lesions. Egypt J Radiol Nucl Med.

[33] Sah VK, Wang L, Min X (2015). Multiparametric MR imaging in diagnosis of chronic prostatitis and its differentiation from prostate cancer. Radiol Infect Dis.

[34] Rhodes DJ, Hruska CB, Conners AL (2015). Journal club: molecular breast imaging at reduced radiation dose for supplemental screening in mammographically dense breasts. AJR Am J Roentgenol.

[35] Gurleyik G, Aktekin A, Aker F (2012). Medical and surgical treatment of idiopathic granulomatous lobular mastitis: a benign inflammatory disease mimicking invasive carcinoma. J Breast Cancer.

[36] Taghizadeh R, Shelley OP, Chew BK (2007). Idiopathic granulomatous mastitis: surgery, treatment, and reconstruction. Breast J.

[37] Yau FM, Macadam SA, Kuusk U (2010). The surgical management of granulomatous mastitis. Ann Plast Surg.

